# Acoustic emission induced by sand liquefaction during vibration loading

**DOI:** 10.1038/s41598-022-21257-6

**Published:** 2022-10-07

**Authors:** Vladimir Frid, Semen Shulov

**Affiliations:** Civil Engineering Department, Sami Shamoon College of Engineering, 84 Jabotinsky St., Ashdod, Israel

**Keywords:** Natural hazards, Solid Earth sciences

## Abstract

The article deals with the study of poorly graded sand samples of different grain content subjected to liquefaction. The research results show the V-shaped behavior of the AE parameters that correspond to the three-stage sand behavior: Phase A is associated with microfractures/displacements between sand grains caused by an increase in pore pressure before the liquefaction point. Phase B (the stage of AE silence just before the liquefaction point) reflects the equality between pore pressure and stress in the confining chamber. Phase C (the stage of increase in AE parameters’ values) is explained by intense friction between sand grains during their movement caused by liquefaction. Our results show that the AE behavior before, at, and after the liquefaction point is significantly affected by the sand grain content. The change in the sand composition from the poorly graded dune sand to "extremely poorly graded sand" significantly increases the time for the creation of the liquefaction state while the coarser the sand grains become, the longer duration of vibration loading is required to reach the liquefaction state.

## Introduction

### Sand liquefaction

The dynamic behavior of soils associated with liquefaction is an important phenomenon in earthquake (EQ) engineering^1–3^. Liquefaction is generally understood as the transformation of granular material from a solid to a liquefied state due to an increase in pore water pressure and reduction in effective stress resulting in a “loss of strength and stiffness in soils”^1^.

A more accurate definition^4,5^ for sandy soils is as follows: for loose sand initial liquefaction is a state of softening in which for an indefinite period suddenly there is a large deformation with an almost complete loss of strength during/immediately after 100% water pressure increase. For sand of medium density to a dense state softening, limited liquefaction, cyclic softening, or cyclic mobility also produced at 100% water pressure growth is accompanied by approximately 5% axial peak-to-peak deformation, but after that, the deformation does not become infinitely large and there is no complete loss of strength in the sample even after the start of the initial liquefaction.

The rate and magnitude of pore pressure generation during EQs have an essential outcome on soil stability^6, 7^. The dynamic behavior of soils is affected by their type, the intensity of applied load, and the degree of saturation^8^. The transformation of the stress–strain to the liquefaction state follows with the intensive reduction in the initial dynamic characteristics of sands^9^ while most soil deformations occur after the start of liquefaction^8^. Soil failure caused by liquefaction often takes time to develop and may occur after the earthquake has ceased^10^. The cyclic tests have been widely used to study the dynamic behavior of soils. For example^11^, the result of dynamic tests on the Niigata sand employed the time histories similar to those of accelerograms recorded at the time of the Niigata earthquake. It was shown that liquefaction could occur 9 s after the initiation of the main shaking and that 20 cycles of load application are enough to reach the liquefaction state. The issue of the time of liquefaction triggering^8^ was studied in the frequency domain while the frequency content of a ground motion changes rapidly upon triggering of liquefaction: namely, high-frequency components are weakened and low-frequency components are amplified upon triggering. The phenomenon was explained due to the changes in soil stiffness that is different before and after the triggering of liquefaction when the soil behaves almost like a liquid^8,12^. Such an accelerogram-based approach was shown to be a promising tool for liquefaction occurrence^12–18^.

The relationship between the liquefaction safety factor and the value of the maximum shear effort applied was established^19^. It was noted that this relationship is affected by the soil density and grain size distribution. The effect of relative density, the intensity of horizontal stress, and the intensity of applied oscillations were studied^20,21^. It was shown that the soil resistance to liquefaction significantly depends on a previous seismic activity^15^.

The main focus of previous researchers^22^ was to demonstrate the importance of achieving 100% saturation of the laboratory sand sample in order to avoid overestimating the strength of the sample against liquefaction. However, the resistance of sands to liquefaction due to partial saturation has been carefully studied by researchers as well over the years^23^. For example, cyclic shear tests indicated that a 10% decrease in soil saturation doubled the liquefaction resistance of medium-dense sand specimens^24^. At full saturation, the highest undrained shear strength ratio causing liquefaction is 0.24 while at partial saturation, the range of B-values that cause "flow" is greater than 0.7 and more than 0.3 for triaxial compression and tension, respectively^25^. The liquefaction analysis of imperfectly saturated soils has led researchers to conclude that a decrease in the degree of saturation leads to more cycles (hence the time with the same frequency of vibration) to achieve liquefaction^23^. The results of the combined study^26^ of Skempton’s B-value and P-wave speed indicated that the velocity of P-wave propagation tends to increase from about 500 m/s to about 1800 m/s when the B-value increases from 0 to 0.95. Similar results were presented in^23^. Note that the motivation for study^26^ was to find the geophysical parameter which can be used in situ instead of Skempton’s B-value (the use of which is non-practical in situ conditions)^26^.

## Acoustic emission

Acoustic emission (AE) is a phenomenon recognized related to frictions between sand grains^27–29^ that is widely applied to study the failure features of various materials including soils, e.g.^30,31^. The AE signals were registered during compaction of coarse-grained sand^21^ and water penetration into granular samples^32,33^. For example^34^, it was shown that the excitation of AE is detected during the onset of the clastic and stiffness recovery regimes^35^. The generation of low-frequency AE components^30^ (below 100 kHz), medium–high-frequency AE components (100–200 kHz), and high-frequency components (200–700 kHz) during sand compression was shown to be induced by the different modes of micro-behavior of the restructuring particles, roughness, abrasion, and microcracks, respectively. It was noted^36^ that in loose sands destroyed by “swelling”, more AE signals were recorded than in densely packed sands destroyed by shear banding. It was also found that the peak frequency of AE fluctuates around the stress peak. A close correspondence was found between the number of AE pulses, their energy, and the stress–strain state of the soil^31^. A concise review of AE caused by failure of soil samples was presented in^37,38^ where the AE phenomenon was investigated using dry and wet sands of different grain content. It was shown that failure of granular materials associated with the AE in the frequency range of tens kHz while low-frequency diapason (< 20 kHz) provides information on the features of the shear zone while the higher range (> 30 kHz)—on stress release events^39,40^. The AE phenomenon induced by soil failure has been studied concerning the stability of slopes, e.g.^41–44^. The analysis of the state of the art portrays that the majority of AE studies during soil compression were performed in the conditions of static/quasi-static loading. The application of AE for the study of granular materials/sands failure associated with liquefaction phenomenon had been performed twenty years ago while up to date is still rare probably due to the complexity of such measurements. AE rate value (the number of AE hits per 10 s of the test) was employed for the study of the change of yield locus^45^. It was shown that the boundary between elastic and plastic behavior significantly changed during compression and extension loading. The undrained cyclic loading (the loading period ~ 900 s, the frequency ~ 1.11 × 10^-3^ Hz) significantly affects the features of elastic–plastic behavior of sand samples and is accompanied by an accumulation of pore pressure^46^.

The analysis of the state-of-the-art shows that: (a) the features of AE before, at, and after the liquefaction state is reached were not studied in detail, especially at a relatively high frequency of vibration (1 Hz), (b) the effect of sand grain size on AE behavior during dynamic loading is still not completely understood yet.

Two approaches to AE measurement are equally and successfully used as is follow: the parametric-based (the classical approach, which allows performing a post-pone statistical analysis of a *big number of AE events*) and signal-based (quantitative which consists of the entire waveform recording with further analysis of a *significant number of AE parameters*)^47^. Both approaches have their pros and cons. Since the use of AE recording in the field of soil mechanics is rare, especially for the study of liquefaction, the AE parameters that are most sensitive to liquefaction are not yet clear. The use of resonant sensors can allow recording the large database of AE signals for statistical analysis (e.g., AE hits). However, some information based on the AE waveform may be lost. That is the reason for the use the signal-based approach in the study.

The present work being based on the triggering of soil liquefaction including the effects of imperfect saturation fills the existing knowledge gap.

## Materials and methods

### Materials

**Figure 1:**
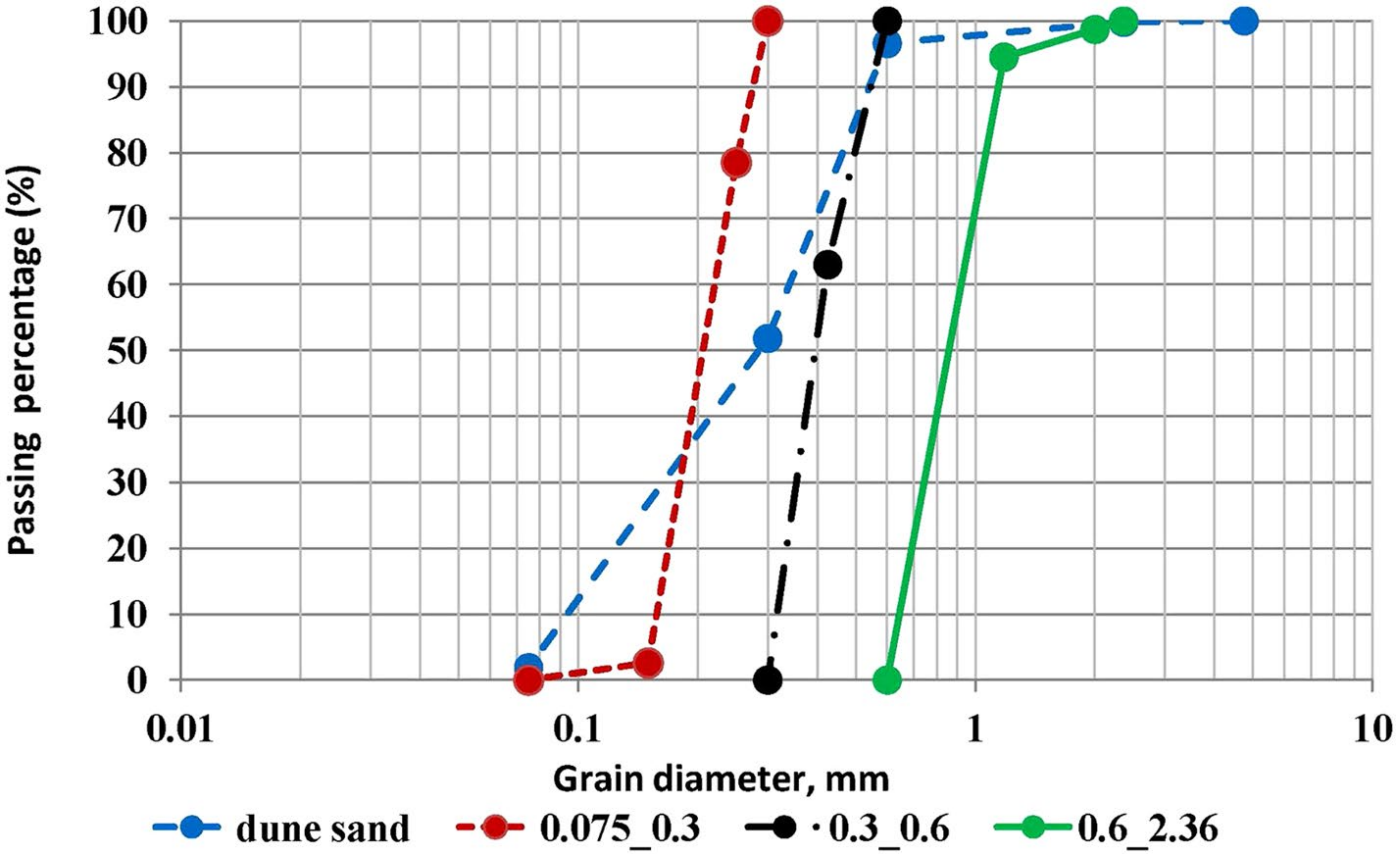
The diagrams of grain distributions of four samples used for the study.

The materials and methods used in the study are described in detail in our previous articles^37,38^, below we will only briefly consider these issues. The dune sand material for the study was collected in the coastal area of ​​Ashdod (about 30 km south of Tel Aviv city, Israel). The standard sieve set was used for the grain size study as follows: 4.75, 2.36, 2, 1.18, 0.6, 0.425, 0.3, 0.25, 0.15 and 0.075 mm. The values of uniformity and curvature coefficients are 1.47 and 0.94, respectively. According to the Uniform Soil Classification System (USCS)^46^, the dune sand has an index of SP (poorly graded sand). It is seen that the grain size diagram of the dune sand looks like a zigzag curve including two break points at 0.15 and 0.6 mm. The diagram was divided into three parts (fine, intermediate, and coarse). The finest part of the dune sand diagram was taken for the study excluding its finest ("Mud") fraction (D-size < 0.075 mm, #200 passing). The low boundary of the most course part was taken at the level of the second break point of the dune sand diagram (D-size > 0.6 mm)—the graph is quite parallel to the X-axis. The boundaries of the intermediate part are 0.3—0.6 mm. The research experiments were conducted with four types of samples: the dune sand and its three fractions sifted as follows: 2.36–0.6, 0.6–0.3, 0.3–0.075 mm. It is seen (Fig. 1—the blue line) that the percentage of noted above three fractions in the content of dune sand is as follows: 5%, 45%, and 50%, respectively. The diagrams of grain distribution for three sifted fractions of dune sand are shown in Fig. 1 as well (in green, black, and red in the order of the decrease in grain size, respectively). The value of D10 for these three fractions is 0.65, 0.3, 0.17 mm (for the fractions of 2.36–0.6, 0.6–0.3, 0.3–0.075 mm, respectively) and for the dune sand is 0.18 mm. D50 for three sifted fractions is 0.2, 0.4, and 0.8 mm, respectively while for the dune sand is 0.3 (Fig. 1). The values of uniformity and curvature coefficients for three sand fractions are as follows: 1.38 and 0.33, 1.33 and 1.02, 1.23 and 0.9, respectively. It is not a surprise that three fractions sifted from the dune sand are classified as SP as well. The values of specific gravity of dune sand, maximum, minimum density, relative density, and density index determined following the standard procedures^48–50^ are as follows: 2.66, 1.89 g/cm^3^, 1.59 g/cm^3^, and 0.7 and 0.67, respectively (see below for the current value of dry density).

### The instruments

#### Loading tools

**Figure 2:**
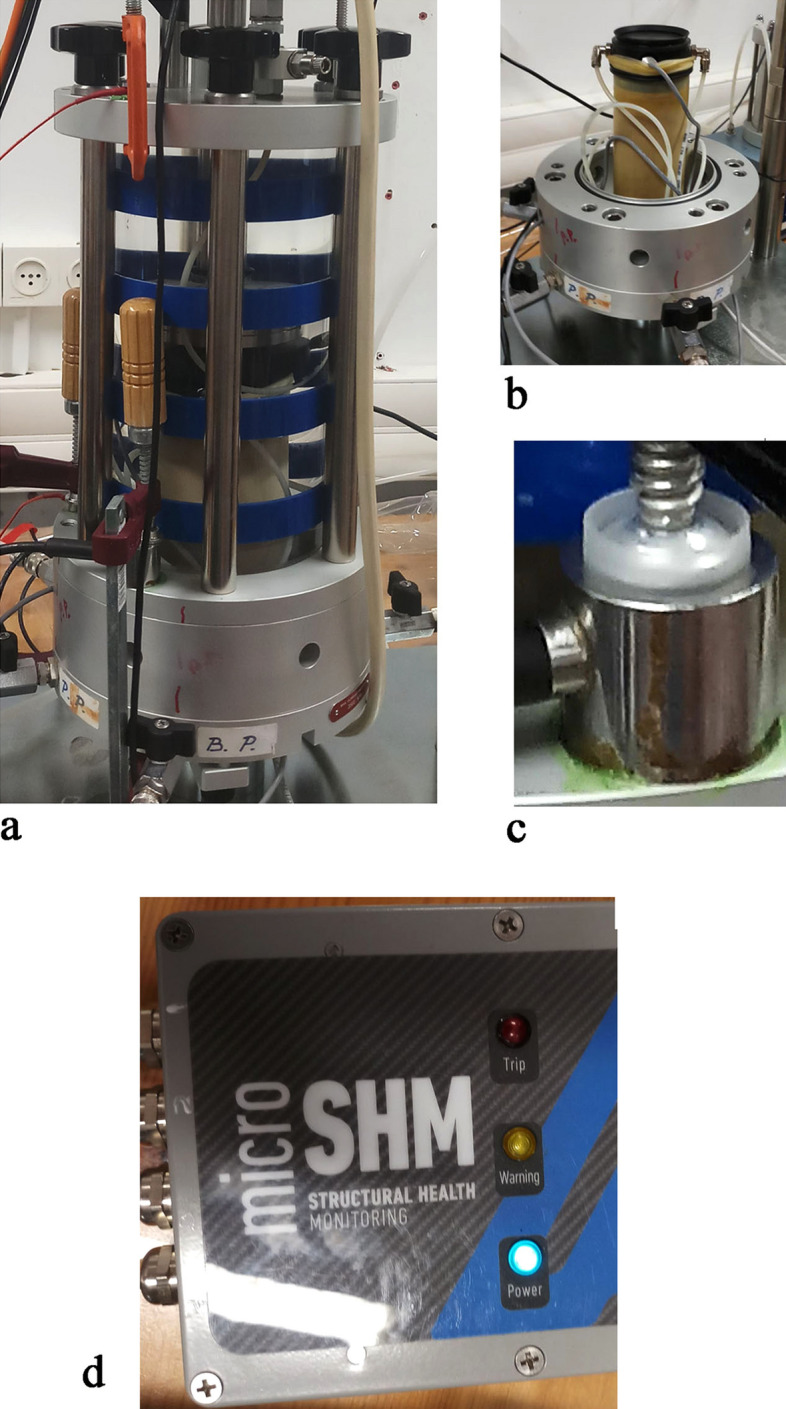
The research instruments: (**a**) the dynamic loading system (Controls SpA., Italy), (**b**) the sand sample: diameter and length are 70 and about 120 mm, respectively, (**c**) the AE sensor. (**d**) The “micro SHM monitoring system” (Mistras/Physical Acoustic Inc.).

The dynamic loading system (Controls SpA., Italy) combined with the AE registration instruments were employed for the experiments (Fig. 2a) in the stress control mode. The parameters of the dynamic loading are as follows: (a) the diameter and length of the sand samples are 70 and about 120 mm, respectively (Fig. 2b), (b) the frequency of sinusoidal vibration—1 Hz, (c) the amplitude of vertical vibrations ± 350 N (± 90 kPa), (d) the value of initial effective confining stress^50^ after isotropic consolidation of all samples (the value of effective confining stress in the confining chamber at the moment of vibration beginning, σ′)—100 kPa), (e) the duration of vibrations—100 s, (f) the initial value of CSR^51^ is 45.0 (the value of reduction factor was neglected).

#### Samples' preparation

The sample preparation procedure for the experiments consisted of six main stages: (a) the raw sand material was dried for a day in an oven at a temperature of 105 °C^52^, (b) the sand was rolled out in a thin layer, immersed in the deaired water in the desiccator for 1 day, the saturated sand was then put into the oven and heated up to 60 °C for several hours being weighted several times till the water content reach the value of 5%.

The value of 5% of the water content is the experimentally found largest amount of water that can be absorbed by a sand sample without squeezing out the water during sample compaction^37,38^, (c) each cylindrical sand sample was prepared inside an elastic jacket/membrane and into a mold on the pedestal (Fig. 2b) using a wet-tamping method^52–54^ by using a hand-held tamper. To ensure homogeneity of the samples they were tamped in eight layers of equal thickness (15 mm each). The initial value of dry density of sand samples prepared for each experiment was 1.79 ± 0.03 g/cm^3^. The value of wet density was of the order of 1.88 ± 0.03 g/cm^3^, (d) the end cup of the sample was connected to a vacuum pump to create a vacuum of 100 kPa in the pore space of the sample, and after vacuumization, the mold was removed and the impermeability (lack of leakage) of the latex membrane was checked. Such a procedure enabled one to avoid sample disturbance by water tubes in the confining chamber. (e) The initial value of water pressure inside the confining chamber was 50 kPa. (f) The sample saturation procedure was performed in the following sequential steps: (f1) following ASTM 5311 requiremnt^51^ the Back Pressure (BP) was increased by 70 kPa, (f2) The Cell Pressure (CP) was increased by 70 kPa, (f3) The check of the increase in the ratio of Pore Pressure ΔPP to increase in Cell Pressure ΔCP (ΔPP/ΔCP) (so-called Skempton’s B-value) was performed, (f4) the procedure was repeated to increase the ratio of ΔPP/ΔCP to ~ 0.8. Note that in the sand liquefaction field, it is accepted to use fully saturated sand specimens (Skempton’s B-value is more than 0.95)^22^ which enables reaching the liquefaction state quite quickly^4,6,55–57^. The less Skempton’s B-value^25^, the more the number of cycles to reach the liquefaction state (for the same test conditions). Since the number of cycles is related to the vibration time through an unchangeable vibration frequency, an imperfect saturation of the sand enables to increase the time to reach the liquefaction state in the sand samples. This extended period of time before the liquefaction point is necessary to properly distinguish between the AE behavior before the liquefaction point (Ru < 1) and at the liquefaction point (Ru = 1) and then to compare both periods with AE behavior just after the liquefaction state is reached.

#### Geomechanical parameters used for the analysis

Typically, a range of geomechanical soil deformation parameters are used to study vibration-induced liquefaction as follows: stress–strain curve, build-up of excess pore water pressure versus a number of cycles, the effective stress path build-up of axial strain versus a number of cycles, etc.^55–58^. To characterize the behavior of the AE before the liquefaction state is reached, at the moment of the liquefaction and after this moment the following geomechanical parameters were studied: (a) the maximum value of excess pore pressure ratio "R_u_" (the ratio of the maximum excess of pore pressure at a given vibration cycle "Δu" to the initial value of effective confining stress in the confining chamber—"σ′_30_" that is equal to 100 kPa—see above), b. The value of deviator stress (q = σ_1 _− σ_3_, where σ_1_ and σ_3_ are vertical and horizontal stress values that the test sample sustains), c. The value of mean effective stress (p′ = (σ_1_ + 2*σ_3_)/3 − Δu, (d) the value of axial strain (ε_a_,%).

#### Acoustic emission

The AE sensor (Fig. 2c) was connected to the “micro SHM monitoring system” (Mistras/Physical Acoustic Inc.) (Fig. 2d) (frequency range—5 kHz–1 MHz) and the sampling rate was 0.2 μs. The 200–800 kHz High-Sensitivity Flat Frequency Response AE Sensor was used for the measurements. The “AE win for Micro SHM” software was used for data continuous acquisition during the entire vibration loading.

**Figure 3:**
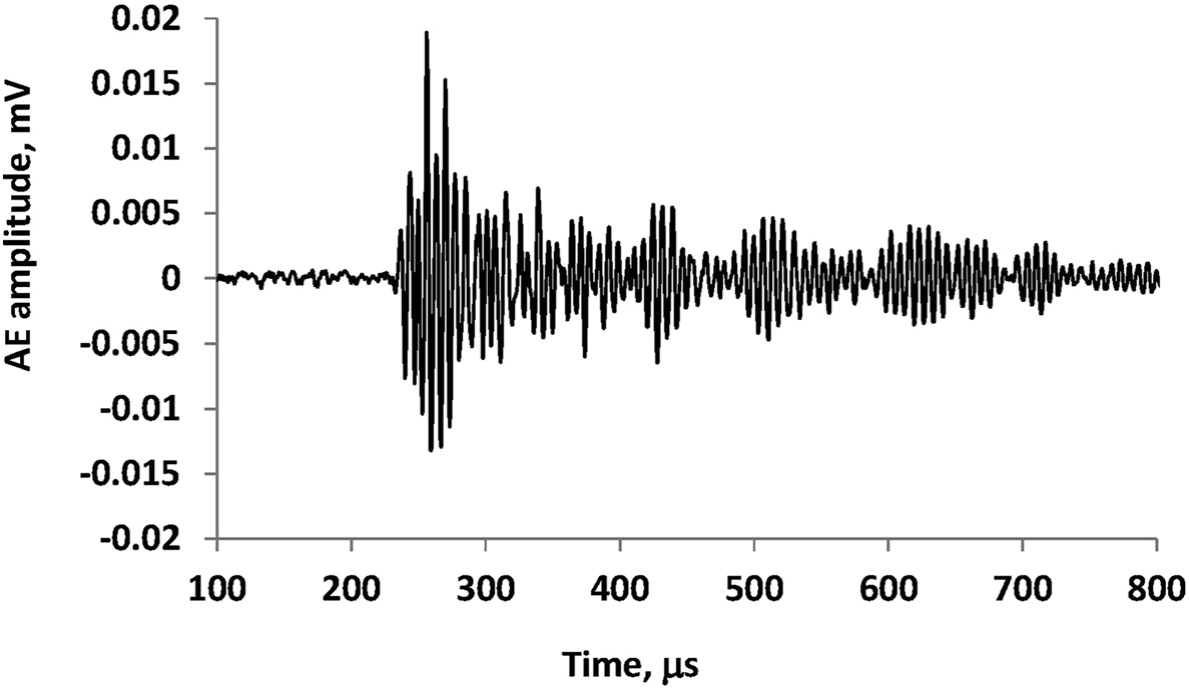
The example of AE hit.

Figure 3 shows a typical example of an AE hit measured during the loading stage.

Eleven parameters of AE hits were investigated as follows: (a) the number of AE hits, (b) the Rate of the number of AE signals—the Time between AE events per Hit Number), (c) the time between the AE signals, d. the RISE-TIME—The time from the first threshold to the peak of the highest waveform (μs), (e) the COUNTS—The number of times the letter AE crosses the identification threshold from the beginning of the letter to the end, (f) the DURATION—The Duration of the AE pulse (μs), (g) the ENERGY—The time integral of the signal voltage, (h) the AMPLITUDE—The maximum value of voltage in the form of a wave AE (AE_db_), (i) the AVERAGE FREQUENCY—The value of counts divided by the length of time (parts 1000—in units of kHz), (j) the PEAK NUMBER (PCNTS)—The number of cycles that exceed the measurement threshold at the time of the rise of AE signal, (k) the ABSOLUTE ENERGY—The time integral of the signal voltage at the sensor before any amplification divided by a 10kΩ impedance and expressed in aJ (attoJoules—10^−18^ Joules).

## Results

### The results of geomechanical studies

**Figure 4:**
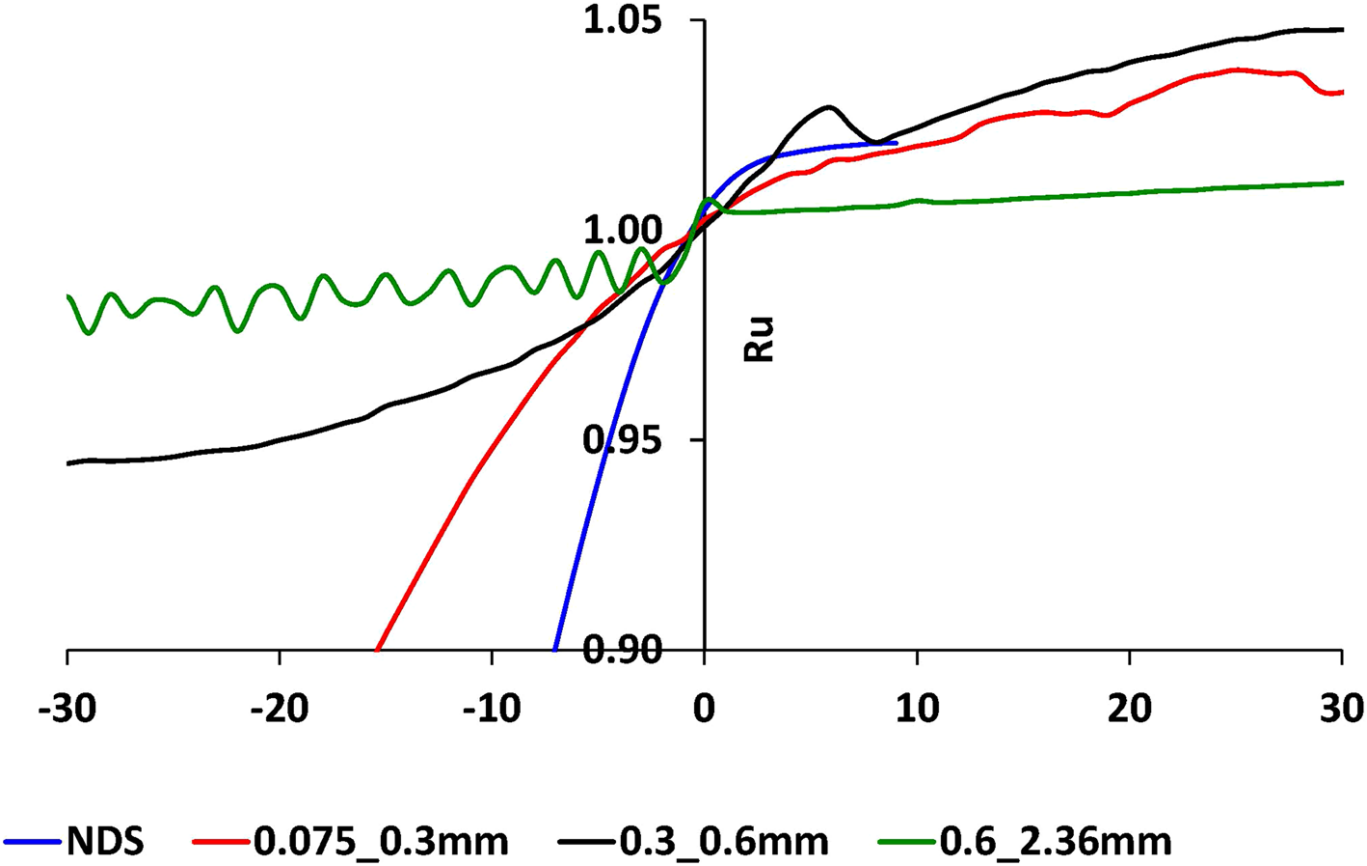
The changes of excess pore pressure vs. the number of vibration cycles N_cyc_ for four sand samples under study, the X-values is the number of vibration cycles while 0 when the R_u_ value is reached to 1 (the negative value of N_cyc_ means the state before the liquefaction point while the positive values after the liquefaction point), the Y-axis is the value of excess pore pressure ratio R_u_. The blue, green, black, and red lines are for natural dune sand, fractions 2.36–0.6, 0.6–0.3, 0.3–0.075 mm, respectively.

Figure 4 shows the changes in the excess pore pressure ratio R_u_ depending on the cycle number N_cyc_. Note that the number of cycles N_cyc_ = 0 corresponds to the case when R_u_ = 1, so if N_cyc_ is negative, the sample is before reaching the liquefaction state, and if N_cyc_ is negative, then the sample is after reaching the liquefaction state. Analysis of Fig. 4 shows that:

- The time needed for the transition of sand to a liquefied state depends on its composition. The shortest time (the highest rate) is set for dunes sand which is a mixture of three other fractions (Sect. “Methods and materials”), the larger the grain size content of sand, the longer it takes to reach the liquefaction state,
- Once the liquefaction mode is created the R_u_ value for all samples is similar. The maximum rate of R_u_ change is for the sample of dune sand (Fig. 4—blue line),
- Approximately 10 s after the creation of the liquefaction state, the value of the rate of change "R_u_" is similar for all studied sand samples.

**Figure 5:**
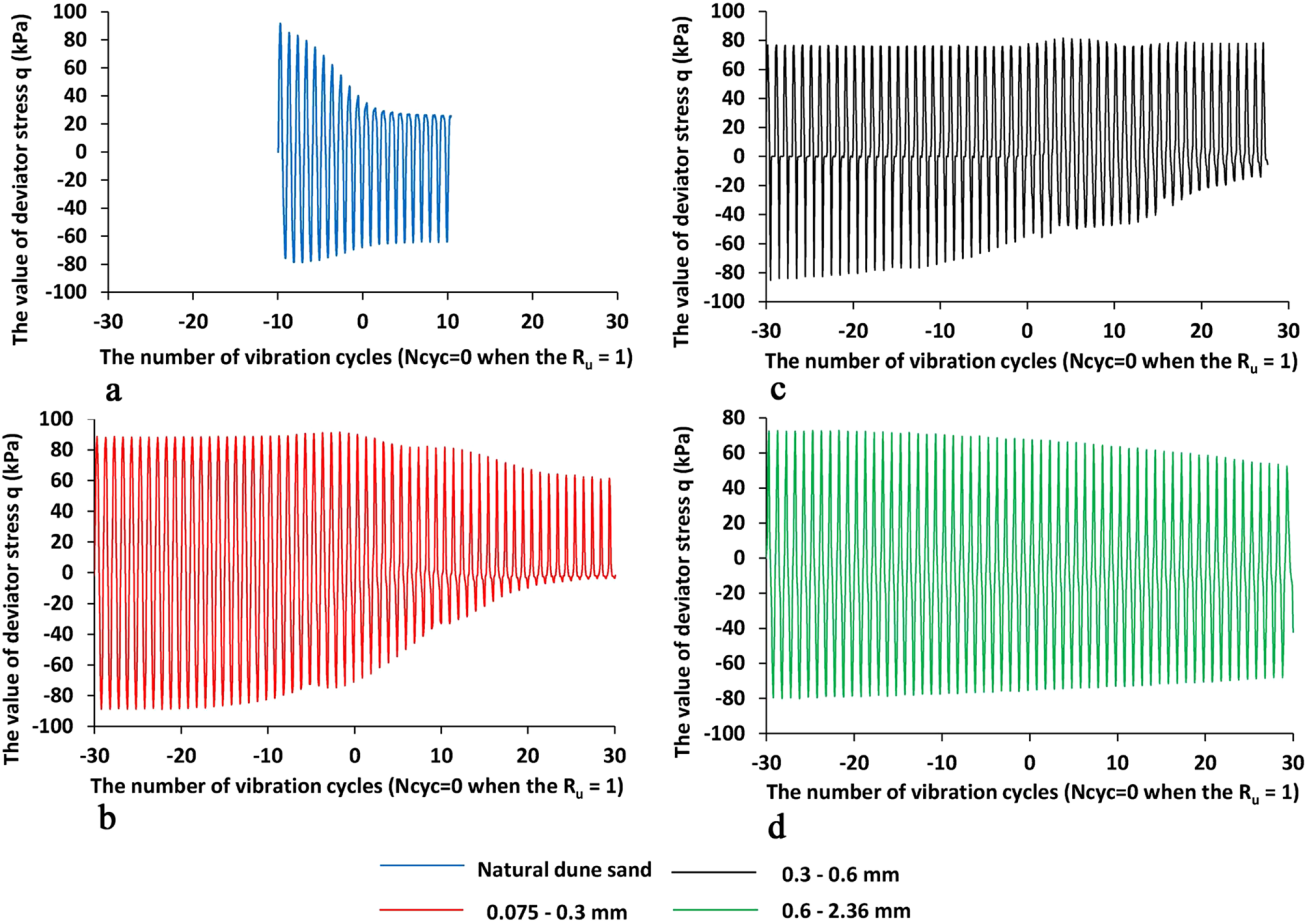
The changes of deviator stress 'q' vs. vs. the number of vibration cycles N_cyc_ for four sand samples under study, the X-values is the number of vibration cycles while 0 when the R_u_ is reached to 1—Fig. 4 (the negative value of N_cyc_ means the state before the liquefaction point while the positive values after the liquefaction point), the Y-axis is the value of deviator stress q (kPa). The blue, green, black, and red lines are for natural dune sand, fractions 2.36–0.6, 0.6–0.3, 0.3–0.075 mm, respectively.

**Figure 6:**
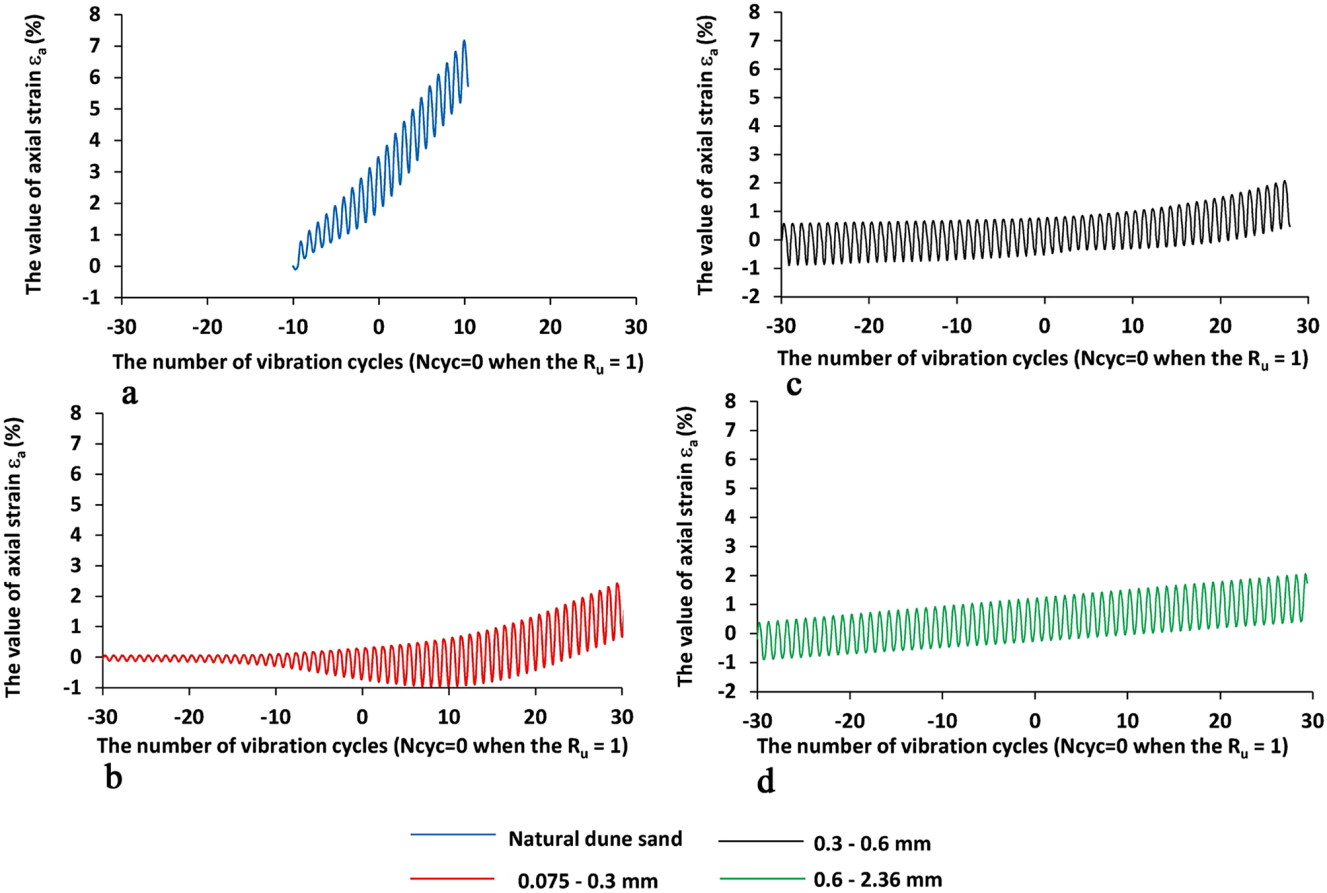
The changes of the value of axial strain ε_a_ (%, positive is compression) vs. the number of vibration cycles N_cyc_ for four sand samples under study, the X-values is the number of vibration cycles while 0 when the R_u_ is reached to 1—Fig. 4 (the negative value of N_cyc_ means the state before the liquefaction point while the positive values after the liquefaction point), the Y-axis is the value of axial strain (%). The blue, green, black, and red lines are for natural dune sand, fractions 2.36–0.6, 0.6–0.3, 0.3–0.075 mm, respectively.

**Figure 7:**
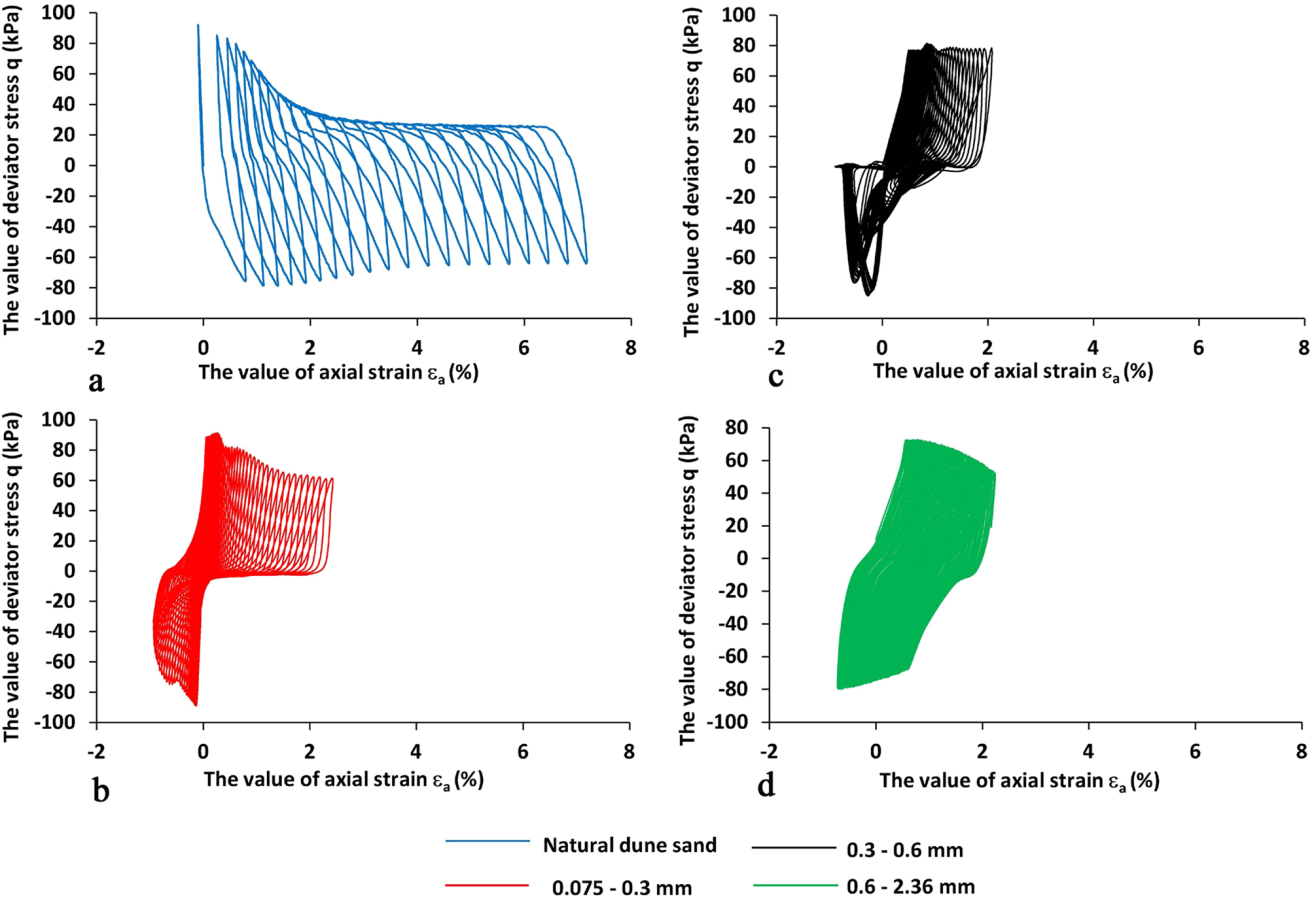
The deviator stress (q, kPa) vs. the value of axial strain ε_a_ (%, positive is compression) number of vibration cycles N_cyc_ for four sand samples under study, the X-values is the value of axial strain ε_a_ (%,), the Y-axis is the value of deviator stress (kPa). The blue, green, black, and red lines are for natural dune sand, fractions 2.36–0.6, 0.6–0.3, 0.3–0.075 mm, respectively.

**Figure 8:**
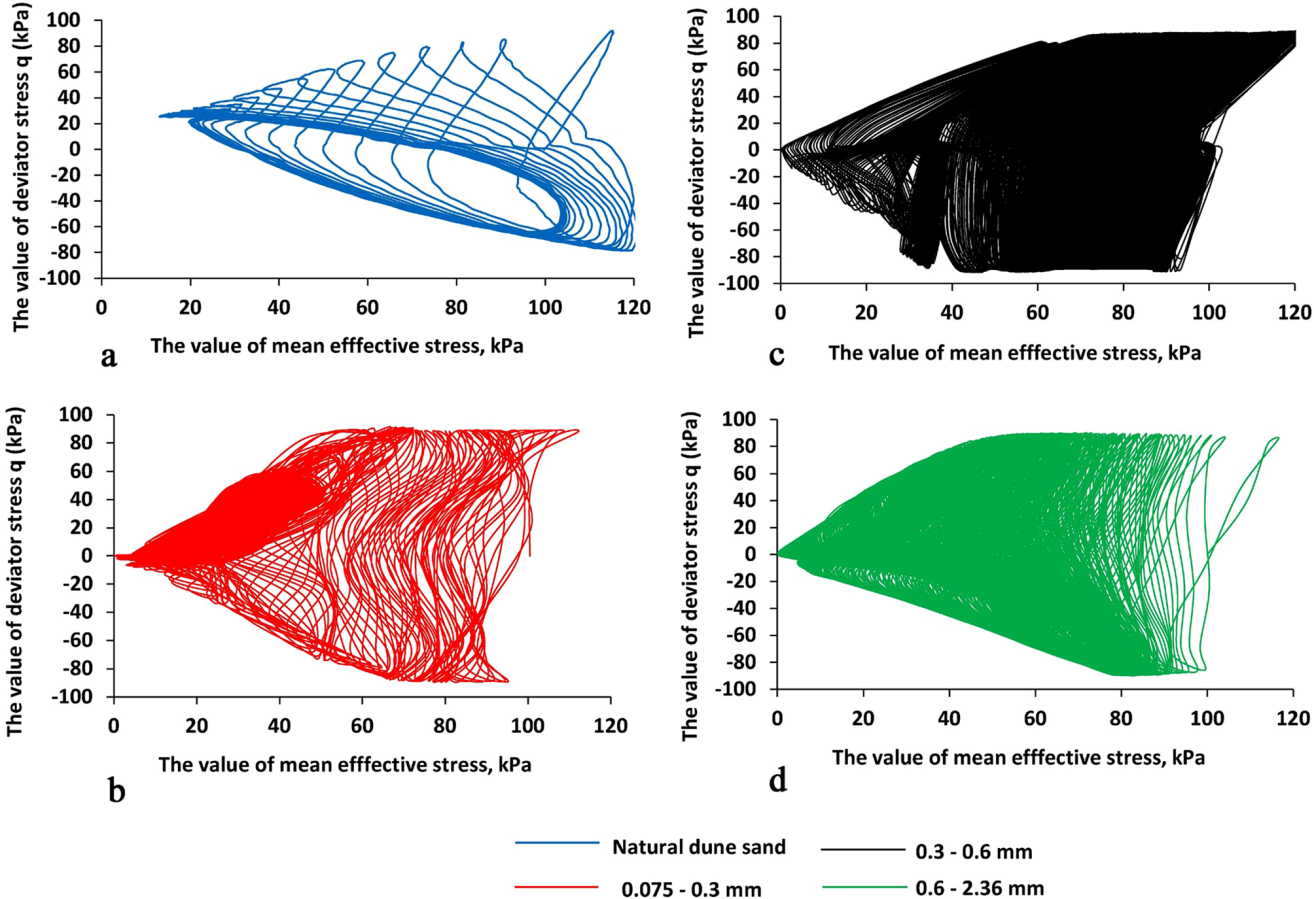
The value of deviator stress (q, kPa) vs. the value of mean effective stress (p′, kPa), the X-axis is the value of mean effective stress (p′, kPa), and the Y-axis is the value of deviator stress (kPa). The blue, green, black, and red lines are for natural dune sand, fractions 2.36–0.6, 0.6–0.3, 0.3–0.075 mm, respectively.

Figures 5 and 6 show the changes in the values of deviator stress 'q' and axial strain ε_a_, respectively, vs. the number of cycles while N_cyc_ = 0 means the value of excess pore pressure ratio R_u_ = 0. Figures 7 and 8 portray the corresponding stress–strain relation ('q' vs. ε_a_) and the stress path ('q' vs. p' ), respectively.

Analysis of Figs. 5, 6, 7 and 8 shows that:

- The change in the deviator stress 'q' compared to the liquefaction point (R_u_ = 1, N_cyc_ = 0) is significantly affected by the composition of the sand. The most significant effect is seen for the natural dune sand. Although it belongs to the SP group, it has the widest (for the four types of sand studied) range of grain sizes (Fig. 1). It can be seen that changes in the q values begin just after the onset of vibration and practically do not change after reaching the state of liquefaction. Comparing the changes in the "q" values of the three screened fractions shows the effect of grain size, namely, the finer the range of grain sizes, the smaller the range of changes in the "q" values. The minimum range of 'q' values falls on the largest fraction (0.6–2.36 mm—green color in Fig. 5d)—the difference in 'q' values between N_cyc_ = − 30 and the liquefaction point (N_cyc_ = 0) is about 6%. An essential seen phenomenon is the asymmetry in the manifestation of 'q' (compression—q > 0 vs. tension—q < 0) for natural dune sand and two sifted fractions (0.075–0.3 mm and 0.3–0.6 mm). It can be seen that the degree and the range of asymmetry decrease with an increase in the size of the fraction.
- The change in the values of axial strain ε_a_ is similar to the change in the values of 'q' compared to the state of liquefaction and grain size (Figs. 6 vs. 5). For example, it is seen that the maximal changes are measured for the natural dune sand (Fig. 6a—in blue). Two essential features of the curve of axial strain for the natural dune sand can be noted: a significant increase in the range of axial deformation during the entire range of vibration and the change in the slope of the curve at the liquefaction point. From the analysis of Fig. 6b–d it can be seen that the increase in the amplitude of axial strain during vibrations and the change in the slope of the curve compared to the liquefaction point also depend on the grain size, namely, the larger the grain size, the smaller the changes.
- The features in sand behavior can be seen in Fig. 7 as follows: (c1) the natural dune sand is the most sensitive to the change in deviator stress, i.e., its hysteresis curve is the widest and the range of axial strain is maximal, (c2) The increment in axial strain (Δε_a_) caused by an increase in the deviator stress 'q' during successive vibration cycles depends on the grain size of three sifted fractions: the larger the grain size, the smaller the increment of axial strain and less its range.
- Figure 8 clearly shows the effect of grain size and increased sensitivity of natural dune sand to vibration compared to the three sifted fractions.

### Results of AE studies

**Figure 9:**
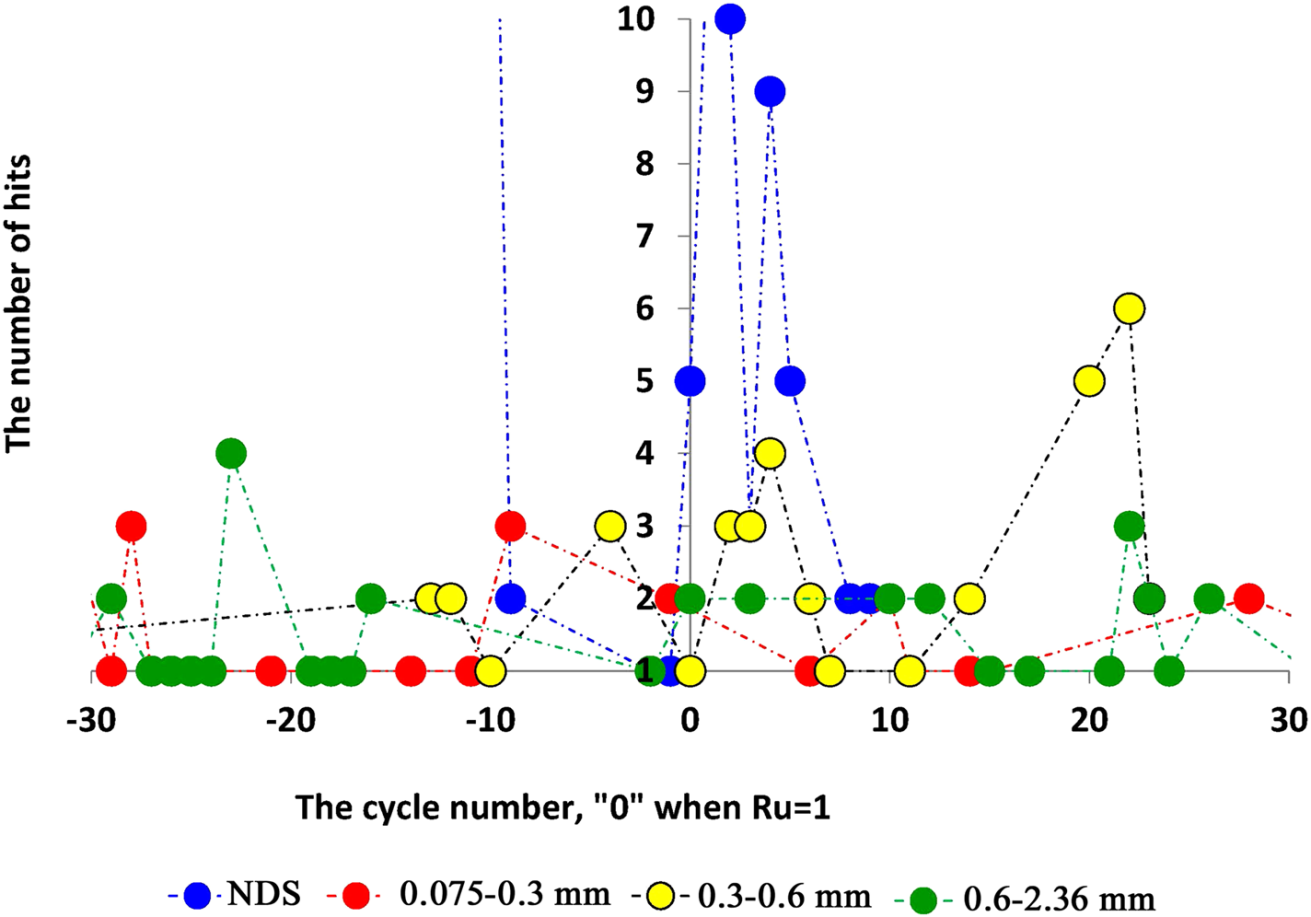
The changes in AE hit number before and after the liquefaction state is created, the X-values is the number of vibration cycles while 0 when the B factor value is reached to 1, the Y-axis is the value of AE hit number. The blue, green, yellow-black, and red circles are for dune sand, fractions 2.36–0.6, 0.6–0.3, 0.3–0.075 mm, respectively.

**Figure 10:**
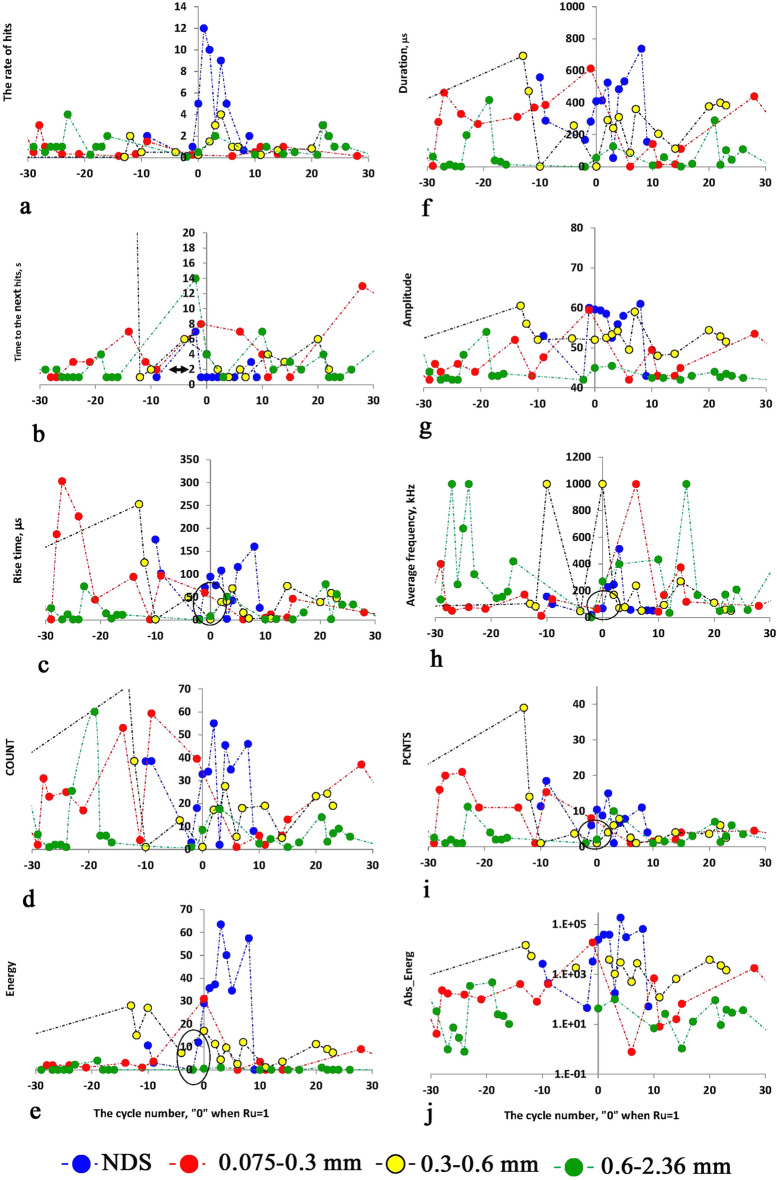
Ten AE parameters are as follows: (**a**) the rate of the number of AE signals (i.e., The Time between AE events per the Hit Number), (**b**) the time between the AE signals, (**c**) the change in the Rise-Time of AE signals, (**d**) the change in Counts, (**e**) the change in energy value, f. The change in signals' duration, (**g**) the change in signals' amplitude, h. The change in signals' frequency, (**i**) the change in the number of peaks in AE signals, (**j**) the change in the value of absolute energy of AE signals, the X-values is the number of vibration cycles while 0 when the B factor value approaches the value equal to 1, the Y-axis is the value of corresponding AE number, the blue, green, yellow-black, and red circles are for dune sand, fractions 2.36–0.6, 0.6–0.3, 0.3–0.075 mm, respectively.

Figure 9 shows the values of AE Hit Number in the range of ± 30 s (N_cyc_ =  ± 30) around the liquefaction point (X-axis: N_cyc_ = 0). It can be seen (Fig. 9) that: (a) the number of AE signals decreases before a liquefaction state is created, (b) there is a certain time range of AE silence (~ 7–10 s, ~ 7–10 cycles before the liquefaction point (N_cyc_ = 0) for all studied samples—see Fig. 10b), (c) the number of AE signals at the liquefaction point increases but it depends on the composition of the sand samples (the maximal value of the AE anomaly is recorded for the natural dune sand, the response of which to changes in the liquefaction state is extremely intense relative to three other fractions studied).

Figure 10a–j show the changes of ten parameters of AE as follows: Fig. 10a—the Rate of the number of AE signals (i.e. the Time between AE events per Hit Number), Fig. 10b—the time between the AE signals, Fig. 10c—the changes in Rise-Time of AE signals, Fig. 10d—the changes in Counts, Fig. 10e—the energy changes, Fig. 10f—the changes in signals' duration, Fig. 10g—the changes in signals' amplitude, Fig. 10h—the changes in signals' frequency, Fig. 10i—the changes in the number of peaks in AE signals, Fig. 10j—the changes in the value of absolute energy of AE signals.

Four essential features of AE anomalies can be noted as follows: (a) the anomaly of AE occurs, usually, a few seconds before the time point of liquefaction is achieved, (b) A certain time before the liquefaction state has been created the value of time between AE hits decreases (Fig. 10b), (c) sometime before the liquefaction state the AE hits disappeared, so-called ”silence time”, (d) immediately before the liquefaction point the time between successive AE hits increases again for all sand fractions. The appearance of the “silence time” probably shows the formation of an equilibrium state between pore pressure and the stress value in the confining chamber (arrow in Fig. 10b). Analysis of Fig. 10c–j portrays that the appearance of all other AE parameters resembles that noted above, namely, their values decrease before the liquefaction point and increase again when the liquefaction state is created. The example of the zone of change in Rise-time behavior is highlighted in the black ellipse in Fig. 10c. Similar behavior can be found for other parameters (e.g., Fig. 10e,h,i). It is seen that some parameters of AE are the most sensitive to the change in the stress state, e.g., the rate of AE hits (Fig. 10a,), the time to the next hit (Fig. 10c), the energy parameters (Fig. 10e,j) and the hit duration (Fig. 10f). Note that this observation is consistent with the results of our earlier studies^37,38^ (see Sect. “Discussion and conclusion”) as well as with the results presented by Hu et al.^43^ and Tanaka and Shirakawa^44,45^.

## Discussion and conclusion

**Figure 11:**
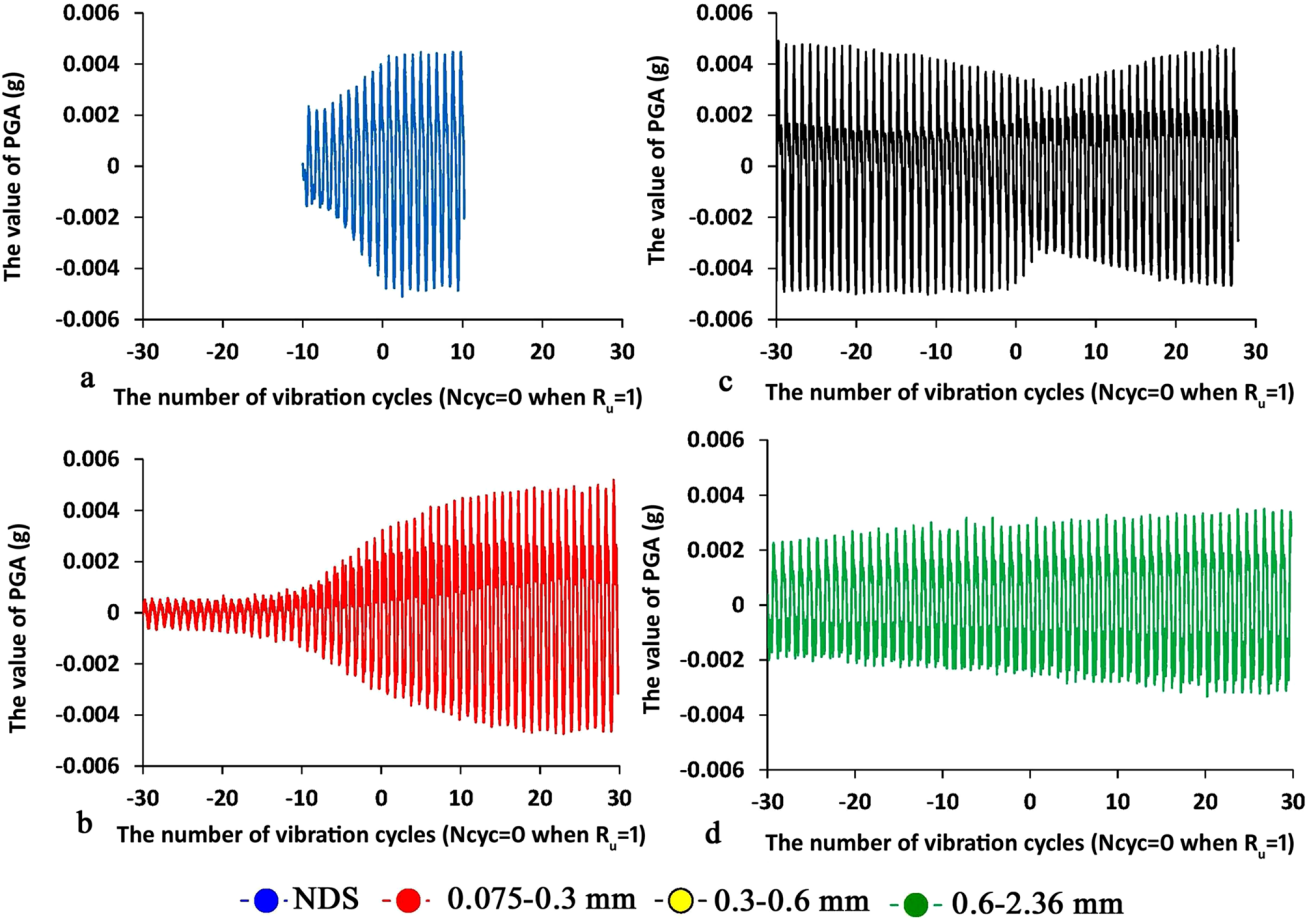
The changes of the value of PGA (g) vs. the number of vibration cycles N_cyc_ for four sand samples under study, the X-values is the number of vibration cycles while 0 when the R_u_ is reached to 1—Fig. 4 (the negative value of N_cyc_ means the state before the liquefaction point while the positive values after the liquefaction point), the PGA values in g units. The blue, green, black, and red lines are for natural dune sand, fractions 2.36–0.6, 0.6–0.3, 0.3–0.075 mm, respectively.

**Figure 12:**
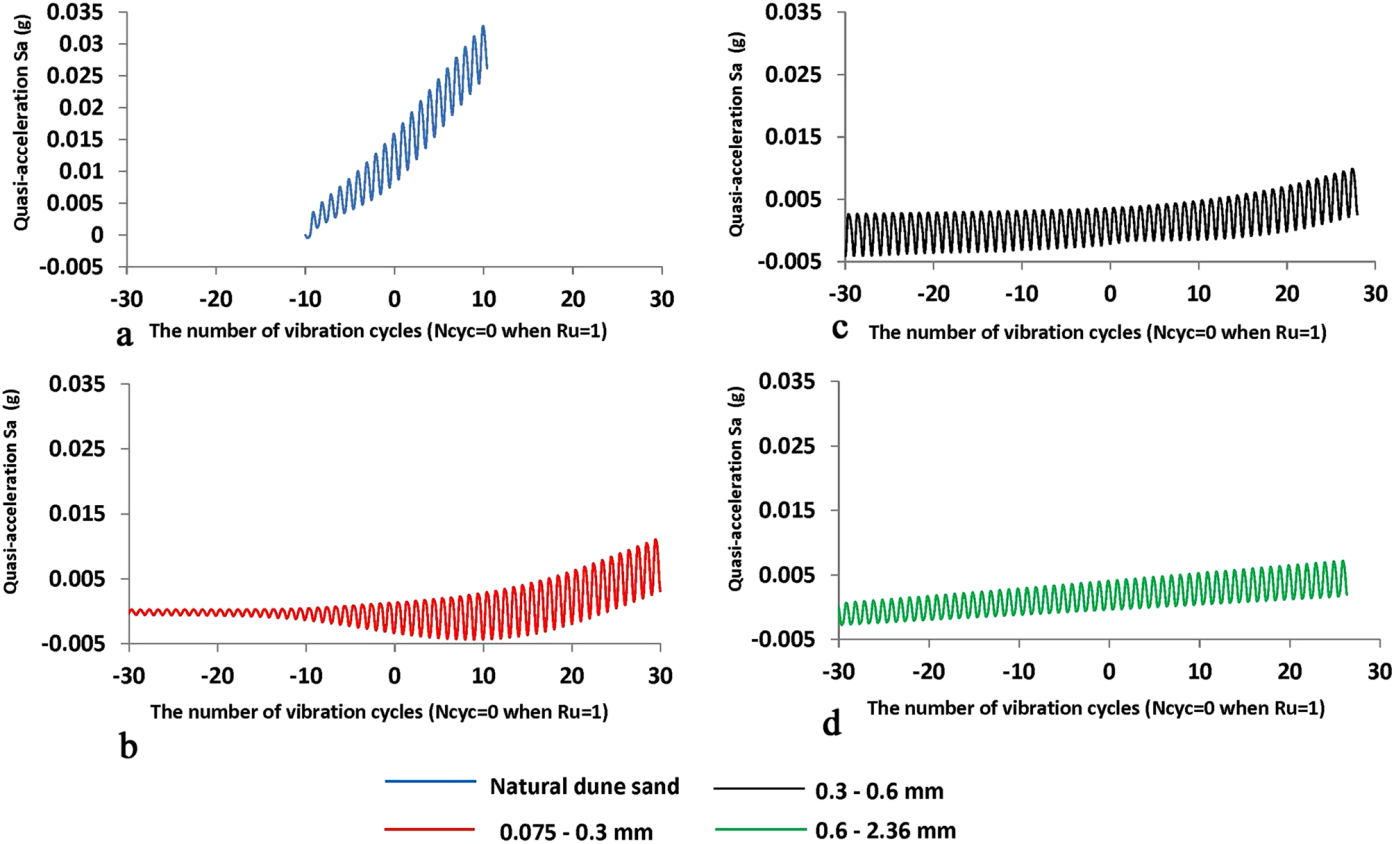
The changes of the value of quasi-acceleration Sa (g) vs. the number of vibration cycles N_cyc_ for four sand samples under study, the X-values is the number of vibration cycles while 0 when the R_u_ is reached to 1—Fig. 4 (the negative value of N_cyc_ means the state before the liquefaction point while the positive values after the liquefaction point), the values of quasi-acceleration in g units. The blue, green, black, and red lines are for natural dune sand, fractions 2.36–0.6, 0.6–0.3, 0.3–0.075 mm, respectively.

Figures 11 and 12 show the values of vibration acceleration (peak ground acceleration—PGA and quasi-acceleration S_a_ in g units (Sa = D*ω^2^, where D and ω are the values of displacement and angular frequency, respectively). The features of the V-shaped behavior of the AE parameters are the most clearly seen in the example of natural dune sand (Figs. 11 and 12a). The acceleration amplitude (Fig. 11a) reaches its maximum value when the liquefaction state is created and does not change significantly after the liquefaction point. The quasi-acceleration S_a_ diagram (Fig. 12a) can be characterized by three key features as follows: (a) the amplitude of the quasi-acceleration increases until a liquefaction state is created while the slope of the graph is still unchanged, (b) the slope of the curve changes when the liquefaction conditions are created, (c) the S_a_ amplitude stays quite unchangeable until the end of the test.

A comparison of three stages (Figs. 11a and 12a) with the three-phase behavior of AE parameters (Figs. 9, 10) portrays that the first stage in Fig. 11a and 12a (the stage of amplitude increase in displacement, velocity, and acceleration corresponds to phase A—the phase of increase in AE parameters value—Figs. 9, 10). The zone before the breaking point in graphs of PGA and S_a_ (Figs. 11a and 12a) nearly corresponds to Phase B where the AE is not caused (so-called the zone of silence, Figs. 9, 10b). Stage 3 (Figs. 11a and 12a) corresponds to Phase C where the magnitude of AE parameters increases again. Note that Stage 3 is the stage of the unchangeable amplitude of PGA and S_a_ and the change in the slope of the S_a_ curve that is much larger than the slope in Stage 1.

It can be assumed that such behavior of AE in phase A is due to microfractures/displacements between sand grains caused by an increase in pore pressure. Phase B reflects the equality between pore pressure and cell pressure. The behavior of AE in phase C can be explained by intense friction between sand grains during their movement caused by liquefaction. Note that, despite the obvious signs of the V-shaped behavior of the AE excitation compared to the liquefaction point, changes in the AE parameters depend on the composition of the sand similarly to the behavior of PGA and S_a_ curves for different fractions. Namely, the envelope slope of oscillation curves for two fine sifted fractions (0.075–0.3 mm—in red and 0.3–0.6 mm—in black) change relatively the liquefaction point (Figs. 11b,c and 12b,c) while much smoother than in Figs. 11a and 12a. Note that after the slope change the amplitude of the oscillation is still quite unchangeable up to the end of vibrations similar to the results for the natural dune sand. Regarding the coarsest fraction (0.6–2.36 mm) the change of the envelope slope is uneven (Figs. 11d and 12d) while the amplitude of oscillation increases slowly but the range of acceleration oscillations is about factor 2 less than for natural dune sand and both fine fractions.

Such a behavior can be explained by the difference in friction between sand grains of varied sizes, which is manifested in the dissimilarity in geo-mechanical performance and as a consequence difference in AE excitation. As can be seen (Figs. 4, 5, 6 and 7), the behavior of the R_u_ ratio, as well as the values of deviator stress, mean effective stress, and axial strain are qualitatively similar. However, the rate of change of these parameters is different for each type of sand and is related to granular composition. The absolute value of the rate increases with *decreasing* the grain size, while the highest value was noted for natural dune sands, which are a mixture of the three studied fractions.

The noted above similarity in behavior of AE parameters (asymmetrical "V" type) implies that the change in the behavior of AE can indeed be an indicator of the approach to the liquefaction state.

Our previous studies^31,32^ showed that most AE parameters are interdependent apart from the lack of the inter-correlation between two parameters: the number of AE hits and the values of absolute energy of AE hits. Such a similar character of the behavior of the studied AE parameters during liquefaction is surprising. On the contrary, it indicates the consistency of the present study with the previous ones^31,32^ conducted during the static loading.

Note the similarity between the values of PGA(g) and Sa (g) presented above parameters and the values of PGA (g) presented in the Earthquake Catalog^59^ (4.4 × 10^−2^–1.2 × 10^−5^).

The results of our vibration experiments with four different sand types show that if the pore pressure exceeds 80% of the value of effective stress, a liquefaction state will inevitably be reached, and the number of vibrations required to reach this state (or in other words, the time required to reach the liquefaction state) is highly dependent on the granular composition of the sand. For example, the time necessary to create a liquefaction state in the dune sand is only 10 s. This finding means that a magnitude 6–7 earthquake will cause liquefaction of dune sand. The above results are consistent with the previous ones^1^. The changes in the sand composition from the poorly graded dune sand to "extremely poorly graded sand" (consisting of a very thin range of grain sizes e.g., 2.36–0.6 mm, 0.6–0.3 mm, and 0.3–0.075 mm) significantly increases the time for the creation of liquefaction state. Moreover, the coarser the sand grains become, the longer duration of vibration loading is required to reach the liquefaction state. Since the pore diameter is often estimated to be 20% of the D10 size^60^ (the grain size corresponding to 10% sieve passing) and since the soil hydraulic conductivity is frequently considered to be related to the value of D10, the increase in D10 value means the increase in soil hydraulic conductivity and hence the longer time required to reach significant pore pressure in the coarser-grained sand. Comparing the results with those presented by Zhu et al.^61^, it looks as if our results contradict their findings: the increase in hydraulic conductivity indicates a less clogged inter-sand void system with the presence of macro pores. Upon shearing, the macro pores are highly to collapse, favoring a contraction. The test specimen normally becomes more liquefiable. The analysis shows that conditions for both studies are essentially different:

- Zhu et al.^61^ presented observations with loose sand with fine additions (D50/d50 ratio ranges 500–1, where D50 and d50 are the grain size diameter for 50% passing for the skeleton and added fractions, respectively). Our findings with the natural dune sand were received for the D50/d50 ratio 0.375–1.5 (fractions 0.6–2.36, 0.3–0.6, and 0.3–0.075 mm—see Fig. 1). The corresponding ratio for three sifted fractions is 1 (as they do not include any additions).
- The content of added materials in^61^ is 5–15% while all added materials are classified as silt or clay. In the presented study the content of the coarsest fraction (0.6–2.36 mm) is 5% while the content of the finest fraction is 50%. The content of silt material in the natural dune sand is less than 1% and it was sifted out from the finest fraction (0.3–0.075 mm).
- The study by Zhu et al.^61^ presented the results with loose sand (the density index is equal to 0) while our study was performed in relatively dense conditions (the density index is equal to 0.67—see above). The dissimilarity between the two types of experiments shows that their results cannot be directly compared. However, in general, their results are consistent. Both our results and those of Zhu et al.^61^ show that the presence of added material within the skeleton of homogeneous sand makes it easier to reach the liquefaction state. It can be seen that natural dune sands, consisting of three fractions, are the most sensitive to the applied vibration relative to three other fractions and the rate to reach the liquefaction state is the fastest in relation to three fractions of homogeneous composition.

Since the time to reach the liquefaction state is related to the duration of the vibrations caused by the earthquake and the composition of the soil at the base of the structure, the above conclusion has the potential to increase the warning time by increasing the time to create the liquefaction state and therefore creating safer conditions for structures built in the marine environment. Changes in the AE parameters observed during the study indicate the possibility of developing an early warning system for the creation of liquefaction conditions at the base of structures in the marine environment, as well as applying the above parameters to assess the integrity of offshore structures due to soil liquefaction.

The circumstances of the influence of the composition of the sand on the symmetry/asymmetry of the letter "V", as well as its amplitude and aperture, are currently not clear and will be studied in further studies.

## Data Availability

All data generated and analyzed during this study are included in the article.
